# Acute disseminated encephalomyelitis following varicella‐zoster virus infection: Case report of effective treated both in clinical symptom and neuroimaging

**DOI:** 10.1002/brb3.1374

**Published:** 2019-07-25

**Authors:** Qi Wang, Li‐Na Cai, Xiang‐Qing Wang

**Affiliations:** ^1^ Department of Neurology Aerospace Center Hospital Beijing China; ^2^ Department of Neurology Chinese People's Liberation Army General Hospital Beijing China

**Keywords:** acute disseminated encephalomyelitis, central nervous system, multiple sclerosis, neuroimaging

## Abstract

**Introduction:**

Acute disseminated encephalomyelitis (ADEM) is an idiopathic inflammatory demyelinating disorder of the central nervous system (CNS). Early treatment is the key for neurological recovery.

**Methods:**

A case of ADEM associated with varicella‐zoster virus infection was presented, in which magnetic resonance imaging (MRI), cerebrospinal fluid (CSF) examinations were included.

**Results:**

Magnetic resonance imaging of the brain revealed multiple hyperintense lesions at the subcortical level on fluid‐attenuated inversion recovery (FLAIR), and MRI of the spinal cord revealed longitudinally segmented hyperintense lesions at the spinal cord on T2‐weighted images. The patient was treated with methylprednisolone and gancyclovir, and had a favorable recovery. Subsequent MRI of the brain and cervical cord showed the previous abnormal hyperintensities had markedly disappeared.

**Conclusion:**

A rare case of ADEM with longitudinal segmented hyperintense lesions at the spinal cord on T2‐weighted images was presented. Excellent response to ADEM treatment with high‐dose steroids was reported resulting in a remarkable neurological recovery. A long‐term follow‐up is needed for prognosis.

## 1 INTRODUCTION

Acute disseminated encephalomyelitis (ADEM) is an idiopathic, uncommon, but treatable disease of the central nervous system (CNS) that mainly affects children and young adults (Lattanzi, Logullo, Di Bella, Silvestrini, & Provinciali, 2014). The disease onset occurs over days to weeks following an antecedent infection or an immunization. The etiopathogenesis of ADEM is suggested to be an immune‐mediated inflammatory demyelination, which is commonly found in the subcortical white matter. Clinical manifestations include sudden onset of neurological impairment, focal or multifocal, generalized seizures, and psychosis. Subtle magnetic resonance imaging (MRI) features of the brain may detect early disseminated CNS demyelination, especially on fluid‐attenuated inversion recovery (FLAIR). Yet, these manifestations are also similar to those in multiple sclerosis or solitary sclerosis (Lattanzi et al., 2014). Current treatments include high doses of steroids. Intravenous immunoglobulins or plasmapheresis is sometimes required. Most patients make a full recovery, whereas a few patients remained neurologically impaired.

## 2 CASE REPORT

**Figure 1:**
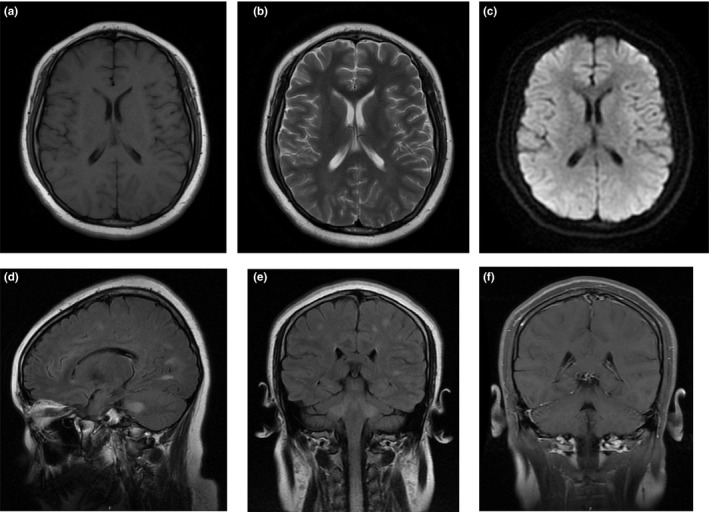
MRI‐scan 3 days after symptom onset. Brain MRI FLAIR image showing areas of hyperintensity involving subcortical white matter of both cerebral hemispheres, pedunculus cerebellaris medius bilaterally, and upper cervical cord. But not obvious on other MRI sequences. (a) Axial T1. (b) Axial T2. (c) Axial DWI (diffusion‐weighted imaging). (d) Sagittal T2‐FLAIR. (e) Coronal T2‐FLAIR. (f) Coronal T1 gadolinium‐enhanced

**Figure 2:**
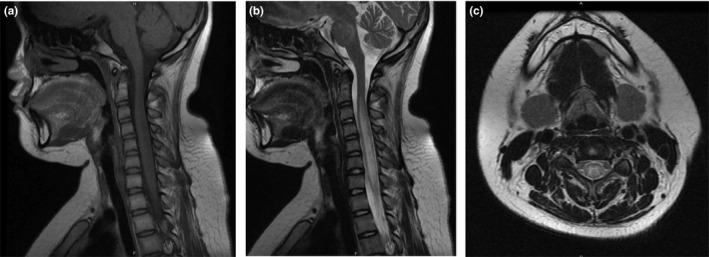
MRI‐scan 4 days after symptom onset. Cervical cord MRI image showing areas of T2 prolongation on the central spinal cord like H, and the cervical cord was swollen. (a) Sagittal T1. (b) Sagittal T2. (c) Axial T2

**Figure 3:**
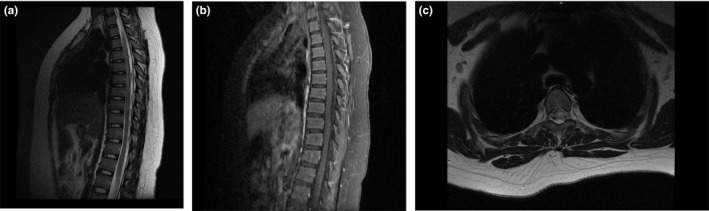
MRI‐scan 4 days after symptom onset. Thoracic cord MRI image showing areas of T2 prolongation in the central spinal cord. (a) Sagittal T2. (b) Sagittal T1 Gd‐enhanced. (c) Axial T2

A 13‐year‐old girl was brought to our hospital with complaints of numbness and weakness of the limbs and urinary retention for 3 days. Eighteen days after the disappearance of measles rashes due to a varicella‐zoster virus infection, the patient felt numbness of limbs, face, and tongue. Gradually, lower limb weakness progressed to both upper limbs. At the same time, she presented with urinary retention. No history of previous virus infection was reported. At early ages, she received routine vaccinations, including poliomyelitis, chickenpox, hepatitis B, and pertussis and no allergies were recorded after vaccinations. Neurological examinations revealed no obvious abnormalities in cortical function. Muscle strength of both upper extremities was grade 4, and grade 2 of both lower extremities. Bilateral tendon reflex was symmetrically brisk. Pinprick sensation was weak below the neck, and vibration sensation was weak below the anterior superior spine. There was dysmetria on finger‐to‐nose tests on both sides of the body. The Hoffmann reflex and Babinski sign were positive on both sides of the body. There were no signs of meningeal irritation. She underwent an MRI of the brain and the spinal cord on the first hospital day (HD) and HD #2 that revealed multiple hyperintense lesions at the subcortical level on FLAIR (Figure 1) and longitudinally segment hyperintense lesions at the spinal cord on T2‐weighted images (Figures 2 and 3). She also underwent a lumbar puncture on HD #1 that revealed pressure was more than 300 mmH_2_O, with cerebrospinal fluid (CSF) containing 200 × 10^6^/L white blood cells of which 95% were mononuclear cells, glucose levels of 2.4 mmol/L, and protein levels of 869.1 mg/L. CMV‐IgG antibody and HSV‐IgG antibody were found positive in serum. Three days of 1,000 mg (patient weight: 80 kg) of intravenous methylprednisolone pulse therapy was given and the dose decreased by half afterward. She was also treated with gancyclovir for a total of 3 weeks.

**Figure 4:**
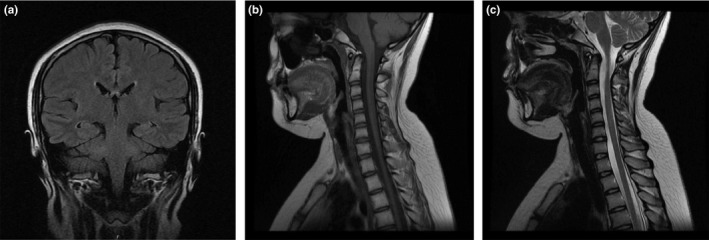
MRI‐scan after treatment. Coronal brain MRI FLAIR image showing no significant change in the appearance of the previous lesions. Cervical cord MRI image showing no significant change in the appearance of the prior lesions, and the appearance of the spinal cord was normal. (a) Coronal brain T2‐FLAIR. (b) Sagittal cervical cord T1. (c) Sagittal cervical cord T2

After treatment with high‐dose steroids for 6 days, her muscle strength of both upper extremities was grade 4+, and grade 3 of both lower extremities. A lumbar puncture was repeated on HD #7, in which pressure was 300 mmH_2_O higher, with CSF containing 40 × 10^6^/L white blood cells of which 95% were mononuclear cells, glucose levels of 3.4 mmol/L, and protein levels of 287.8 mg/L. Serum CMV‐IgG and HSV‐IgG antibodies were negative. In addition, CSF oligoclonal bands (OCB) were positive, whereas myelin basic protein, aquaporin 4 (AQP‐4) antibody, and N‐methyl‐D‐aspartate receptor antibody were negative. Gradually, her sensorium became better, and she was independent in her activities, except urinary retention. No apparent changes in lesions were found in MRIs of the brain and cervical cord on HD #17 and HD #20 (see Figure 4). After continued steroid treatment, we removed her urethral catheter safely on HD #21. She was subsequently discharged on HD #22 on a prolonged oral steroid tapering for 4 weeks. Further improvements and full strength of the limbs were found after 3 months of outpatient follow‐up.

## 3 DISCUSSION

Acute disseminated encephalomyelitis is an acute, sometimes subacute, multifocal, immune‐mediated, monophasic inflammatory disease of the central nervous system (CNS; Wender, 2011). Molecular mimicry associated with infectious agents or direct inflammatory damage to myelinated neurons is a possible mechanism (Marin & Callen, 2013). The brain and spinal cord may appear normal or be congestion with swelling; however, the disease manifests microscopically including perivenous sleeves of demyelination with mild perivascular inflammation. Clinical presentation is characterized by acute encephalopathy, seizures, multifocal neurologic abnormalities, and meningeal signs.

In this report, we presented a case of ADEM in which the patient had acute transverse myelitis according to neuroimaging. Overall in ADEM cases, spinal cord involvement is relatively common and has been described in 11%–28% of children (Tenembaum, Chamoles, & Fejerman, 2002). In a cohort of 176 adults, spinal cord involvement was reported in 83% of patients (Marchioni et al., 2013). It is unclear whether cases only involved the spinal cord can diagnosed as an ADEM variant.

Magnetic resonance imaging of the brain is critical for the differential diagnosis of ADEM. The most common MRI finding is multifocal lesions predominantly involving white matter. Abnormalities are more readily visible on FLAIR images than T1‐ and T2‐weighted images. Studies reported that sagittal scans rarely revealed extended longitudinal lesion beyond one spinal segment (Tillema & Pirko, 2013). A contiguous lesion over three vertebral segments was considered a longitudinally extensive transverse myelitis (Pandit, 2009) in neuromyelitis optica. Relapse of longitudinal lesions was reported to be high (Weinshenker et al., 2006) and for lesions that extend to seven segments were poor (Carnero Contentti et al., 2017). ADEM typically involves the thalamus and/or basal ganglia, but not in multiple sclerosis (MS) that it can be used as a differential diagnosis (Dale et al., 2000). Supratentorial lesions in ADEM tend to be asymmetrical, whereas symmetrical lesions were found in the thalamus and basal ganglia (Tenembaum et al., 2002). A recent study found that, in adults, lesions in the putamen were associated with ADEM whereas neuromyelitis optica (NMO) lesions were commonly found in the hypothalamus (Zhang et al., 2014). ADEM lesions in the brainstem were often bilateral and symmetrical (Lu et al., 2011).

We observed inflammation with pleocytosis and/or elevated protein in CSF in the ADEM patient. It is necessary to exclude acute CNS infection. OCBs in the CSF can also be found, but this is less common than in patients with MS. Children account for 64%–95% of MS patients, but only 0%–29% of ADEM patients (Dale et al., 2000). Several studies have identified AQP4 antibodies in a subset of ADEM patients (Okumura et al., 2015). The presence of CMV and HSV‐IgG antibodies in the serum suggested the potential contribution of these viruses to ADEM. Despite the rarity of the association, increased antibody levels to these viruses in the CSF, as well as diffuse lesions similar to ADEM (Mohsen, Abu Zeinah, Elsotouhy, & Mohamed, 2013; Zaguri, Shelef, Ifergan, & Almog, 2009) in MRI of brain and cervical spinal cord were reported.

Treatment of ADEM includes high doses of steroids. Immunomodulation has been reviewed with satisfactory clinical outcome in both adults and children (Alexander & Murthy, 2011; Dale et al., 2000; Hynson et al., 2001; Straub, Chofflon, & Delavelle, 1997; Wingerchuk, 2008). First‐line treatment usually consists of IV methylprednisolone (20–30 mg kg^−1^ day^−1^), generally 3–5 days with a maximum dose of 1 g/day for both adults and children. An oral steroid taper for 4–6 weeks commonly follows the use of IV steroids, depending on the resolution of clinical symptoms. For patients who do not respond to the initial IV steroid dose, IV immunoglobulin is used as a secondary treatment. Plasmapheresis with 3–7 exchanges is another treatment option, and should be considered early, but little evidence exists for its effectiveness.

Acute disseminated encephalomyelitis is usually a monophasic illness, but recurrent patients have been reported. This patient had an acute onset with only one clinical episode, thus it was acute and monophasic. In case of recurrence in future follow‐up, other diagnoses were considered, such as multiphasic ADEM or recurrent ADEM. There are two recurrent forms of ADEM. Multiphasic ADEM is a recurrent form that involves new brain sites not previously affected, whereas recurrent ADEM has a tendency to involve the previously affected brain region (Cohen et al., 2001). When multiphasic ADEM is diagnosed, a differential diagnosis of MS needs to be considered. Diagnosis of MS is currently based on temporal and spatial dissemination according to the McDonald 2010 criteria. New lesions or relapses after 6 months may indicate the development of MS (Schwarz, Mohr, Knauth, Wildemann, & Storch‐Hagenlocher, 2001). However, the criteria to predict the prognosis of patients with ADEM are limited. Studies suggested scores to predict the response to treatment and disease course in patients with multiple sclerosis (Lattanzi et al., 2015; Rio et al., 2014). A long‐term follow‐up is warranted.

## 4 CONCLUSIONS

Acute disseminated encephalomyelitis rarely presents longitudinally with segmented hyperintense lesions at the spinal cord on T2‐weighted images. With limited case reports, the prognosis is unknown. Excellent response to ADEM treatment with high‐dose steroids was reported resulting in a remarkable neurological recovery. A long‐term follow‐up is needed for prognosis.

## CONFLICT OF INTEREST

The authors declare that they have no conflict of interest.

## DATA AVAILABILITY STATEMENT

All data from this study are included in the article.
